# Structural network efficiency predicts cognitive decline in cerebral small vessel disease

**DOI:** 10.1016/j.nicl.2020.102325

**Published:** 2020-06-25

**Authors:** Esther M. Boot, Esther MC van Leijsen, Mayra I. Bergkamp, Roy P.C. Kessels, David G. Norris, Frank-Erik de Leeuw, Anil M. Tuladhar

**Affiliations:** a Radboud University Medical Center, Donders Institute for Brain, Cognition and Behaviour, Department of Neurology, Nijmegen, the Netherlands; b Radboud University Medical Center, Department of Medical Psychology, Donders Institute for Brain, Cognition and Behaviour, Radboud University, Nijmegen, the Netherlands; c Donders Institute for Brain, Cognition and Behaviour, Radboud University, Nijmegen, the Netherlands; d Erwin L. Hahn Institute for Magnetic Resonance Imaging, University of Duisburg-Essen, Essen, Germany; e Faculty of Science and Technology, Magnetic Detection and Imaging, University Twente, Enschede, the Netherlands

**Keywords:** Small vessel disease, Diffusion tensor imaging, Graph theory, Network efficiency, Cognitive function

## Abstract

Cerebral small vessel disease (SVD) is a common disease in older adults and a major contributor to vascular cognitive impairment and dementia. White matter network damage is a potentially important mechanism by which SVD causes cognitive impairment. Earlier studies showed that a higher degree of white matter network damage, indicated by lower global efficiency (a graph-theory measure assessing efficiency of network information transfer), was associated with lower scores on cognitive performance independent of MRI markers for SVD. However, it is unknown whether this global efficiency index is the strongest predictor for cognitive impairment, as there is a wide range of network measures. Here, we investigate which network measure is the most informative in explaining baseline cognitive performance and decline over a period of 8.7 years in SVD. We used data from the Radboud University Nijmegen Diffusion tensor and MRI Cohort (RUN DMC), which included 436 participants without dementia (65.2 ± 8.8 years) but with evidence of SVD on neuroimaging. Binarized and weighted structural brain networks were reconstructed using diffusion tensor imaging and deterministic streamlining. Using graph-theory, we calculated 21 global network measures and performed linear regression analyses, elastic net analysis and linear mixed effect models to compare these measures. All analyses were adjusted for potential confounders (age, sex, educational level, depressive symptoms and conventional SVD MRI-markers (e.g. white matter hyperintensities (WMH), lacunes of presumed vascular origin and microbleeds). The elastic net analyses showed that, at baseline, global efficiency had the strongest association with cognitive index (CI), while characteristic path length showed the strongest association with psychomotor speed (PMS) and memory. Binary local efficiency showed the strongest association with attention & executive function (A&EF). In addition, linear mixed-effect models demonstrated that baseline global efficiency predicts decline in CI (χ2(1) = 8.18, p = 0.004),PMS (χ2(1) = 7.75, p = 0.005), memory (χ2(1) = 27.28, p = 0.000) over time and that binary local efficiency predicts decline in A&EF (χ2(1) = 8.66, p = 0.003) over time. Our results suggest that among all network measures, network efficiency measures, i.e. global efficiency and local efficiency, are the strongest predictors for cognitive functions at cross-sectional level and also predict faster cognitive decline in SVD, which is in line with earlier findings. These findings suggests that in our study sample network efficiency measures are the most suitable surrogate markers for cognitive performance in patients with cerebral SVD among all network measures and MRI markers, and play a key role in the genesis of cognitive decline in SVD.

## Highlights

• Baseline network efficiency was the strongest predictor for cognitive function in SVD.
• Baseline network efficiency predicted faster cognitive decline in SVD.
• Network efficiency is the most sensitive marker for cognitive function in SVD.
• Network efficiency plays a key role in the genesis of cognitive decline in SVD.

## 1 Introduction

Cerebral small vessel disease (SVD) is prevalent in older adults and can be visualized on neuroimaging as white matter hyperintensities (WMH), lacunes of presumed vascular origin and microbleeds (Wardlaw et al., 2013). SVD is the most impairment cause of vascular cognitive impairment and vascular dementia (Ter Telgte et al., 2018). SVD is a spectrum disorder with each individual suffering from a unique combination of symptoms and there is a wide variability of disease progression. While some patients show a benign disease course, other deteriorates rapidly (Ter Telgte et al., 2018). The underlying neural mechanism of cognitive impairment in SVD is however still not completely understood, as these MRI markers for SVD are generally weakly associated with or do not explain the full variance of cognitive impairment. A potential important mechanism by which SVD causes cognitive impairment is the disruption of the white matter network in SVD, as evidenced by recent studies (Lawrence et al., 2014, Kim et al., 2015, Tuladhar et al., 2016, Lawrence et al., 2018).

In recent cross-sectional studies, the associations between conventional MRI markers of SVD and cognitive impairment were, at least in part, mediated through white-matter network damage assessed by structural network efficiency(Reijmer et al., 2013, Lawrence et al., 2014, Kim et al., 2015, Tuladhar et al., 2016, Lawrence et al., 2018). Furthermore, structural network efficiency predicted conversion to dementia in SVD independent of conventional MRI markers of SVD (Tuladhar et al., 2016, Lawrence et al., 2018). These studies suggest that network efficiency measures based on structural networks might be used as a disease marker in SVD.

Network measures can be calculated using graph theory, a mathematical model to assess the network topology quantitatively (Bullmore and Sporns, 2009, Sporns, 2014). One such graph-theoretical measure is the structural network efficiency, that is, global efficiency, which has been commonly used in previous studies in SVD patients (Lawrence et al., 2014, Tuladhar et al., 2016, Tuladhar et al., 2016, Lawrence et al., 2018). Global efficiency quantifies the extent to which information communication is globally integrated in a network. This global information communication is topologically disrupted due to the SVD-related white matter damage. Intuitively, the more damage to the white matter the less efficient white matter network (i.e. less integration of information communication globally in a network). Global efficiency is thought to be the most sensitive marker as disease marker in SVD, due to the widespread damage in SVD (Ter Telgte et al., 2018). However, other network measures, such as measures of centrality and rich club organization are also associated with cognitive impairment in SVD (Reijmer et al., 2016, Tuladhar et al., 2017). These measures are related to connections central to global brain communication and integration of a network. Furthermore, measures of segregation (e.g. clustering coefficient, transivity and modularity) (Kim et al., 2015b), which refer to the network’s ability for specialized processing to occur within interconnected groups of brain regions, are also linked to cognitive performance in SVD. To date, it remains unknown which network measure is the most informative and most predictive with regard to cognitive performance in SVD among all network measures.

In this study, we aim to investigate which network measure is the most predictive in explaining cognitive impairment in SVD, independent of conventional MRI markers of SVD and whether this network measure at baseline predicts cognitive decline over time. We hypothesized that a network efficiency measure, e.g. global efficiency, that captures the cumulative effects of SVD-related lesions might be most predictive in explaining the cognitive performance at cross-sectional level and cognitive decline in SVD compared to other network efficiency measures.

## 2 Material & methods

### 2.1 Participants

This study is a part of the Radboud University Nijmegen Diffusion tensor and MRI Cohort (RUN DMC) study (van Norden et al., 2011). All participants, aged between 50 and 85 years, with SVD on neuroimaging, which was defined as presence of WMH or lacunes, were included between October 2002 and November 2006. Detailed information about the recruitment of the study sample can be found elsewhere (van Norden et al., 2011). The MRI was scheduled within weeks after the visit to the research center. At baseline neuroimaging and neuropsychological testing was available for 503 patients. We excluded additionally 67 patients due to either territorial infarcts, inadequate MRI quality and failure of the imaging processing pipeline, yielding a total sample of 436 participants with neuroimaging at baseline. Follow-up was available for 434 participants in 2011 (5.3 years between first and second time-point) and 308 participants in 2015 (8.7 years between first and third time-point).

### 2.2 Standard protocol approvals, registrations, and patient consents

All participants gave written informed consent according to the Declaration of Helsinki. The medical ethics committee region Arnhem–Nijmegen approved the study.

### 2.3 Cognitive testing

All participants underwent extensive neuropsychological testing at 3 time-points over a period of 8.7 years. Detailed information has been published previously (van Norden et al., 2011). We transformed the raw test scores to z-scores and used thematically similar tests to calculate cognitive performance. In this study we included the cognitive index (CI), psychomotor speed (PMS), memory and attention-executive function (A&EF). CI is a compound score calculated as the mean of the z-scores of: 1) the one-letter subtask of the Paper-and–Pencil Memory Scanning task, 2) the reading subtask of the abbreviated Stroop Color-Word Test, 3) the Modified Symbol–Digit Substitution Task, and 4) the mean of the total score on the three learning trials and the delayed recall of the 3-trial Rey Auditory Verbal Learning Test (RAVLT) (de Groot et al., 2000). We calculated PMS as the mean of the z-scores of 1) the one-letter subtask of the Paper–and-Pencil Memory Scanning Task, 2) the reading subtask of the abbreviated Stroop Color-Word test, and 3) the symbol–digit substitution task. For memory, we calculated the mean of the z-scores of the immediate and delayed recall of the Rey Auditory Verbal Learning Test (RAVLT) and the Rey Complex Figure Task (RCFT), as well as Speed–Accuracy Trade-Off (SAT) scores of the 2- and 3-letter subtasks of the Paper-Pencil Memory Scanning Task (PPMST). We calculated A&EF as the compound score of 1) the interference score of the Stroop Test by dividing SAT-scores of the color-word task by the mean SAT-scores of the reading and color naming tasks of the Stroop Test 2) the mean z-score of the verbal fluency task, and the mean z-score of the SAT-scores of the Verbal Series Attention Test (VSAT). We calculated cognitive decline for each participant individually, by subtracting baseline scores from the follow-up scores.

### 2.4 Other measurements

We used the Center of Epidemiological Studies on Depression scale (CES-D) to assess depressive symptoms. We defined hypertension as systolic blood pressure ≥140 mm Hg or diastolic blood pressure ≥90 mm Hg or use of antihypertensive drugs. Blood pressures were measured 3 times in supine position after 5 min of rest. The use of antidiabetic or lipid-lowering drugs was used to assess whether the participant was affected by diabetes or hypercholesterolemia. Body mass index (BMI) was calculated as weight (in kg) divided by height (in m) squared. We obtained the smoking status through standardized questionnaires, which was checked during the interview. Co-morbidities were collected by structured questionnaires, except for blood pressure, length and weight which were measured during the consultation. The questionnaires were checked during the consultation.

### 2.5 MRI data acquisition

Due to the scanner upgrade between 2006 and 2011, we were unable to combine the baseline and follow-up MRI data. Therefore, we used the baseline MRI data obtained in 2006. The baseline MRI data was acquired on a 1.5-Tesla MRI (2006; Siemens, Magneton Sonata). The protocol included a T1 3D magnetization-prepared rapid gradient echo imaging (repetition time [TR] = 2.25 s, echo time [TE] = 3.68 ms, inversion time [TI] = 850 ms, flip angle [FA] = 15°, voxel size 1.0 × 1.0 × 1.0 mm), a fluid-attenuated inversion recovery (FLAIR) sequence (2006: TR = 9.00 s, TE = 84 ms, TI = 2.20 s, voxel size 0.5 × 0.5 × 0.5 mm, plus an interslice gap of 1 mm), T2*-weighted gradient echo sequences (TR = 800 ms, TE = 26 ms, voxel size 1.3 × 1.0 × 6.0 mm, interslice gap 1 mm), and a DTI sequence (TR = 10.10 s, TE = 93 ms, voxel size 2.5 × 2.5 × 2.5 mm, 4 unweighted scans, 30 diffusion-weighted scans with b value of 900 s/mm2).

### 2.6 MRI markers of SVD

MRI markers of SVD were assessed according to the Standards for Reporting Vascular changes on neuroimaging (STRIVE) (Wardlaw et al., 2013). WMH were manually segmented on FLAIR images by two trained raters blinded to clinical information. The WMH volume was calculated by summing the segmented WMH multiplied by the slice thickness. Lacunes and microbleeds were manually assessed by a trained raters. The interrater and intrarater variability were good with weighted kappa values of 0.87 and 0.95 for the presence of lacunes, and 0.85 and 0.86 for the presence of microbleeds. Interrater variability (assessed by intraclass correlation coefficient) for total WMH volume was 0.99 (Tuladhar et al., 2016a). We used Statistical Parametric Mapping (SPM12) for segmentation on T1 images to obtain gray matter (GM), white matter (WM), and cerebrospinal fluid (CSF) probability maps. These maps were binarized and summed to supply total volumes. The GM, WM and CSF volumes were calculated by adding the values from the respected tissue class image (e.g. indicating the probability of a given class) and multiplying by the volume of each voxel. To adjust for head size, these volumes and WMH volume were normalized to the total intracranial volume. We calculated the mean fractional anisotropy (FA) and mean diffusivity (MD) within the whole white matter using DTI data. We used b0-images to compute the coregistration parameters to the skull-stripped T1-images using Functional MRI of the Brain linear image registration tool (FLIRT). The white matter masks, obtained from the segmentation procedures were then linearly registered to the diffusion space. We then calculated the FA and MD within the white matter.

### 2.7 White matter network construction

We defined network nodes by using the Automated Anatomical Labeling (AAL) template (Tzourio-Mazoyer et al., 2002), which resulted in 90 regions; 45 per hemisphere, excluding cerebellar regions. Using the FSL 5.0.5 tools (Smith et al., 2004), the skull-stripped T1 images were nonlinearly registered to Montreal Neurological Institute (MNI) 152 template using Functional MRI of the Brain nonlinear registration tool (FNIRT). Next, we derived the transformation matrix from the registration of b0-images to T1 participant space using Functional MRI of the brain linear image registration tool (FLIRT). For the FLIRT registration we used a rigid body registration with 6 degrees of freedom. We used these transformations to register the AAL image to each participant’s diffusion image space. Next, we used the in-house developed algorithm named “PATCH” on the raw diffusion data to correct for cardiac and head motion artifacts and eddy currents (Zwiers, 2010). The Diffusion Toolkit (www.trackvis.org) was used to calculate the diffusion tensor and fractional anisotropy (FA). Fiber assignment by continuous tracking (FACT) was used to generate the fiber tracks of the entire brain for each participant. The tracking algorithm started at the center of the voxels with fractional anisotropy >0.2 and ended when the fiber tracks left the brain mask, encountered voxels with fractional anisotropy <0.2 or when the turning angle exceeded 60°. Two regions were considered connected if the endpoints of the reconstructed streamline lay within both regions. The weight of the connection (e.g. the connection strength) was defined as mean fractional anisotropy for each reconstructed streamline multiplied by the number of reconstructed streamlines connecting two regions (Tuladhar et al., 2015). Next, we further normalized the connection strength by the volume of AAL region to correct for the differences in the AAL regions size and the differences in brain size (Brown et al., 2011). This resulted in an undirected, weighted 90 × 90 matrix for each participant.

### 2.8 Graph theory analysis

We assessed graph-theoretical network measures using the Brain Connectivity Toolbox (Rubinov and Sporns, 2010). We calculated 21 different network measures based on binarized and weighted structural networks. The connection strengths in the binarized structural networks are not weighted and represent 0 (absence) or 1 (presence) in the network. These networks are therefore simpler to characterize. In contrast, the connection strengths in the weighted structural networks are weighted as described in the previous section. These networks contain complementary aspects of network organization and seem to be useful in filtering the influence of weak and potentially non-significant connections (Rubinov and Sporns, 2010). The definitions of the different network measures can be found in the *supplement*.

### 2.9 Statistical analysis

To identify the most influential network measure, we used elastic net method with α set at 0.05 to identify the most influential network measure using glmnet R package. The tuning parameter was selected through a 10-fold cross-validation. Elastic net analysis is a sparse modeling method, which addresses high dimensionality and multi-collinearity (Zou and Hastie, 2005), which makes it more reliable than multiple linear regression. This is necessary as several network measures strongly correlated with each other. Network measure that showed the strongest association was selected for further analyses. To test whether selected network measure can predict changes in cognitive performance over time, we fitted linear mixed effects (LME) models with cognitive performance as the dependent variable, indicated as cognitive index and psychomotor speed, using “lme4” version 1.1–14 in R (Bates, 2015). All analyses were performed separately for cognitive index and psychomotor speed. LME models allow us to examine cognitive changes in the entire population while controlling for individual differences in rates of cognitive changes. Unstructured covariance was applied. We fitted two models: (1) a null model including baseline age, sex, education, depression, WMH volume, number of lacunes, number of microbleeds, total brain volume, selected network measure at baseline and time. Subject‐specific random intercept and slope were included in the model; (2) a full model, in which we added an interaction term between selected network measure and time, to investigate whether the selected network measure is related to the rate of cognitive decline over time. We compared model fit between the two models by performing the likelihood ratio test.

### 2.10 Data availability

The data that support the findings of this study are available from the corresponding author, upon reasonable request.

## 3 Results

### 3.1 Descriptive statistics

Demographic and clinical measurements for all 436 participants are reported in Table 1. The mean age was 65.2 years (SD = 8.8) and 45.6% were women. Hypertension was reported in 316 (72.5%) participants. The whole brain networks of all participants had a small-world architecture (combination of high levels of local clustering among nodes of a network and short paths between all nodes) (Bullmore and Sporns, 2009), with a normalized local efficiency >1 and normalized global efficiency <1.

**Table 1:** Descriptive statistics for the study population.

Characteristics	n = 436
*Demographic characteristic*	
Age, years (SD)	65.2 (8.8)
Woman (%)	199 (45.6)
Education, score (range)	5 (1–7)
MMSE, score (range)	29 (27–30)
*Vascular risk factors*	
Hypertension, number (%)	316 (72.5)
Diabetes mellitus, number (%)	302 (69.3)
Use of lipid-lowering drugs, number (%)	191 (43.8)
BMI, kg/m2 (SD)	27.1 (4.2)
Smoking status	
Never, number (%)	134 (30.7)
Former, number (%)	236 (54.1)
Current, number (%)	66 (15.1)
*Neuroimaging characteristics*	
Total brain volume, ml (SD)	1097.8 (120.8)
WM volume, ml (SD)	467.1 (65.8)
WMH volume, ml (range)	6.4 (3.3–16.7)
Lacune(s) (%)	97 (22.3)
Microbleed(s) (%)	67 (15.4)
Mean FA of WM (SD)	0.40 (0.03)
Mean MD of WM, mm^2^⋅ s^−1^ × 10^−3^ (SD)	0.0009 (0.0001)
*Network properties*	
Normalized global efficiency (SD)	0.91 (0.04)
Normalized local efficiency (SD)	3.20 (0.59)

Values represent mean (standard deviation), median (range), or presence (%). Education: 1 being less than primary school and 7 reflecting an academic degree. Lacune(s) or microbleed(s) represent number (percentage) of patients with one of more lacunes or microbleeds on MRI. MMSE: Mini-Mental State Examination. WM: white matter. WMH: white matter hyperintensities.

### 3.2 Graph-theoretical measures are related to cognitive performance

**Fig. 1:**
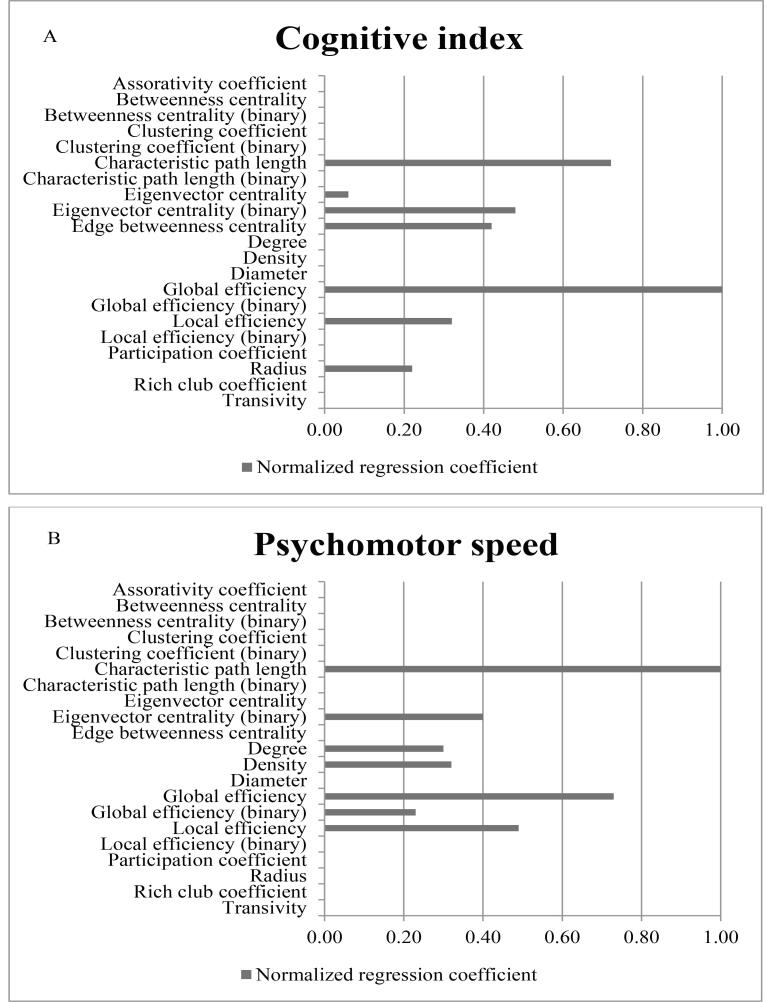

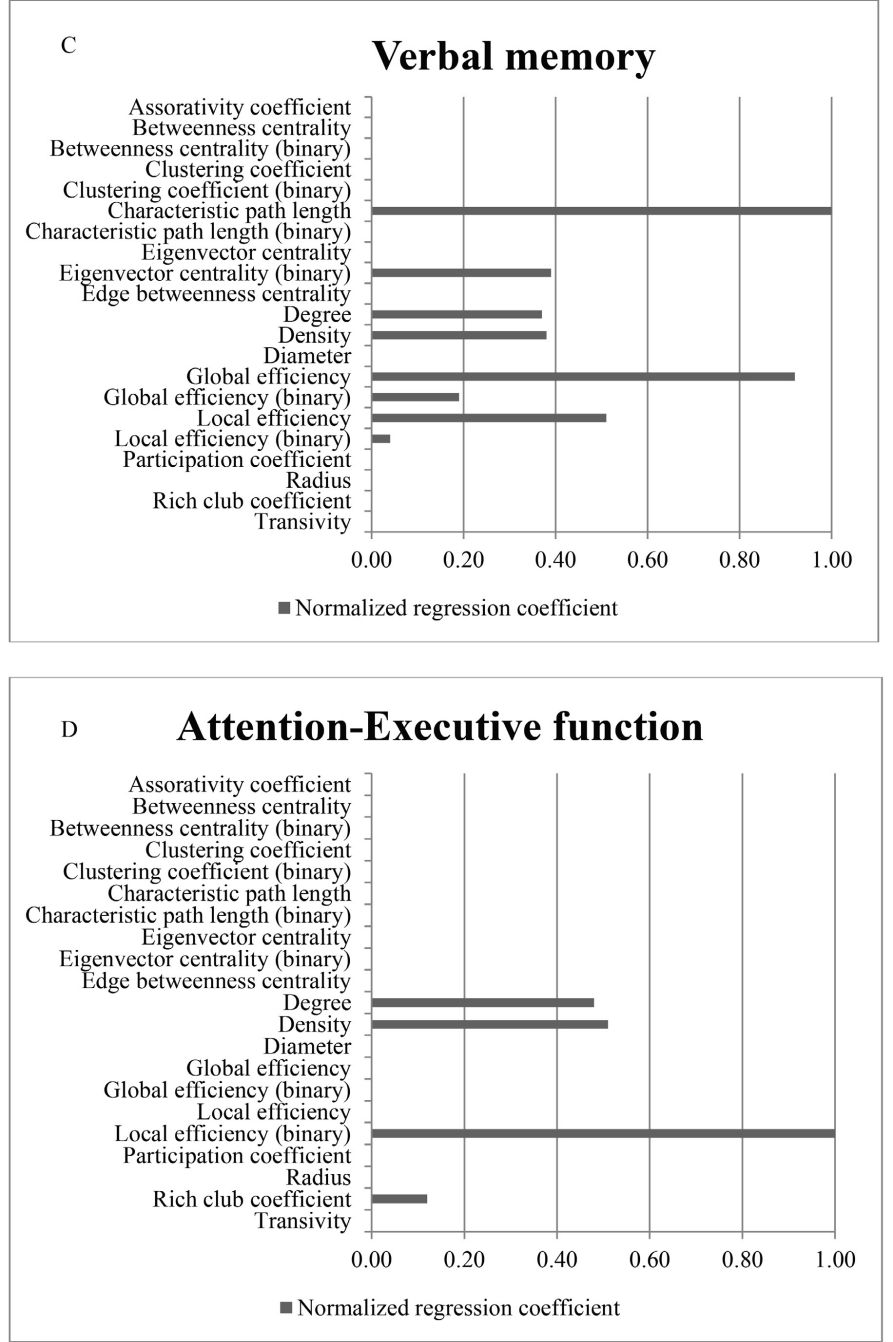
Regression coefficients from elastic net analysis. Represents normalized regression coefficients from the elastic net analysis for cognitive index (A), psychomotorspeed (B), Memory (C) and Attention- Executive function (D), adjusted for age, gender, education, depressive symptoms, WMH volume, presence of lacunes and microbleeds, total brain volume (TBV). Higher coefficients represent stronger association.

The elastic net analysis is shown in Fig. 1. Using elastic net analysis, among all network measures global efficiency showed the strongest association with CI in SVD. Characteristic path length showed the strongest association with PMS and memory followed by global efficiency (normalized regression coefficient was 0.68 and 0.92 respectively). For A&EF, binary local efficiency showed the strongest association. For further analyses, we focused on global efficiency and binary local efficiency, since these network measures seem biologically most informative for cognitive impairment in SVD, whereas characteristic path length is mathematically more complex to use in disconnected nodes (approximately 5% of the study population had one or more disconnected nodes).

### 3.3 Network efficiency is associated with higher rate of cognitive decline over time.

Linear mixed effect models demonstrated that lower baseline global efficiency is associated with greater decline in CI (β = 0.04, 95%CI: 0.01–0.07, p = 0.004), in PMS (β = 0.04, 95%CI: 0.01–0.06, p = 0.005) and in memory (β = 0.08, 95%CI:0.06–0.09 , p = 0.00) over time, independent of the traditional MRI markers of SVD. The full model showed stronger fit when the interaction term ‘global efficiency × time’ was added to the model for CI (χ^2^(1) = 8.18, p = 0.004). Similar findings were found for PMS (χ^2^(1) = 7.75, p = 0.005) and memory (χ^2^(1) = 27.28, p = 0.000). The linear mixed effect model for lower baseline binary local efficiency showed associations with a greater decline in A&EF (β = 0.08, 95%CI: 0.05–0.11, p = 0.003), the full model showed stronger fit after we added the interaction term ‘binary local efficiency × time’ (χ^2^(1) = 8.66, p = 0.003).

## 4 Discussion

In this study, we investigated which structural network measures derived from DTI showed the highest validity in predicting cognitive performance and is associated with cognitive decline over 9 years in SVD. We showed that baseline efficiency measures, e.g. global efficiency, characteristic pathlength and binary local efficiency, showed the highest predictive strength for cognitive performance, indicated by an overall CI and the domains PMS, memory and A&EF. In addition, we demonstrated that the baseline efficiency measures, global efficiency and binary local efficiency, predicted the rate of cognitive change over time. SVD affects the white matter tracts and leads to disconnection between to brain regions, consequently resulting in clinical symptoms. Therefore, it can be seen as a disconnection-syndrome (Ter Telgte et al., 2018).

These findings converge previous cross-sectional MRI studies showing that global efficiency is associated with overall cognitive impairment (Kim et al., 2015, Kim et al., 2015, Reijmer et al., 2015, Tuladhar et al., 2016). Our results suggest that attention-executive functions are more dependent of local network processes, and therefore more associated with local efficiency, whereas the other the cognitive domains (PMS and memory) seems to be more dependent of global processes in the brain, and therefore more associated with global efficiency. For PMS and memory, we found that characteristic path length was the strongest predictor, followed by global efficiency. Characteristic path length refers to the average shortest path length between all pairs of nodes in the network (Rubinov and Sporns, 2010), whereas efficiency is inversely related to characteristic path length. The presence of disconnected nodes, meaning there is no connection to the nodes, will diverge to an infinite characteristic path length. To avoid this, efficiency measure can be used, which is inversely related to characteristic path length. Disconnected nodes will represent a zero efficiency (Rubinov and Sporns, 2010). Therefore, global efficiency is more reliable than characteristic path length in SVD when disconnected nodes are present in the network.

Our results suggest that the assessment of structural network using DTI and tractography can be used to identify further cognitive decline in SVD. We showed using linear mixed model that baseline global efficiency and binary local efficiency predicted faster cognitive decline in SVD independent of MRI markers of SVD. This is in line with the longitudinal MRI studies showing that decline in structural networks predicts dementia in SVD (Tuladhar et al., 2016, Lawrence et al., 2018). In contrast, the conventional MRI markers at baseline were unrelated to cognitive decline in these individuals (van Uden et al., 2015). One possible explanation could be that network efficiency can be seen as a representation of brain reserve (Stern et al., 2018). Patients with high network efficiency are able to better compensate when white-matter disruptions occur by using alternative connections, and are thereby less affected by SVD-related lesions. In contrast, participants with reduced network efficiency may be more vulnerable for further network breakdown and therefore attendant cognitive decline.

Strengths of this study are its large sample size, the extensive neuropsychological testing and a study population that represents the whole spectrum of sporadic SVD. The use of elastic net modeling for investigating which network measure is most informative for cognitive impairment in SVD since it overcomes, unlike multiple linear regressions, the problem high-dimensional data and collinearity between the different network measures (Zou and Hastie, 2005, Reijmer et al., 2015). Furthermore, the use of linear mixed modeling for the longitudinal cognitive data with a follow-up duration of approximately-nine years provides stronger evidence for the relationship between baseline global efficiency and cognitive decline over time.

A number of limitations are recognized. First, this study cohort consists of cognitively relatively healthy participants as participants with severe cognitive impairment (e.g. dementia) were excluded from this study. If any, this may have lead to an underestimation of the effects of the network measures. In later stages of SVD, more SVD-related lesions are observed. We hypothesize that this leads to more white matter network disruption and therefore network measures might be of more importance. Second, the optimal protocol for reconstructing structural networks is still disputed. In this study, DTI data were acquired in 2006 at 1.5 Tesla MRI with relatively low number of diffusion encoding directions, which prevents us from using newer tractography techniques that accounts for among others crossing fibers. FACT streamlining was used to identify white matter tracks for the structural network, which is a computational inexpensive method and therefore easily to apply. Potential limitations of this method are failure in reconstructing white matter tracks in a complex white matter architecture (Mori and van Zijl, 2002), identifying long distance fibers due to noise and partial volume effects (Zalesky and Fornito, 2009). The consistency with other studies suggest that network measures based on deterministic tractography provide biologically meaningful information, though these findings should be verified and replicated with state-of-the art DTI protocols and sophisticated tractography techniques. Additionally, many graph theoretical measures are based on the number of edges and the density of the network. However it remains unclear whether and how corrections should be done for these metrics. Nevertheless, despite that network efficiency measures might be affected by these simple network measures, our results show that network efficiency measures contains biologically important information regarding cognition in SVD. Furthermore, in this study, we included baseline MRI acquired in 2006 and were unable to investigate the change in network measures due to the scanner upgrade between 2006 and 2011. Therefore, we were unable to investigate the relation between changes in network measures over time with regard to cognitive decline. Another limitation is the attrition bias in our study, which should be taking into account when interpreting the results. Participants could not be included in the analysis due to death, being lost to follow-up, or not having cognitive data available at follow-up, all of which is practically unavoidable in longitudinal study. Participants who were not included were older, performed worse on cognitive testing at baseline and had a higher SVD load.

In conclusion, we demonstrated that the network efficiency measures, global efficiency and (binary) local efficiency, are the strongest predictor for cognitive performance in SVD among all network measures and are associated with cognitive decline. These findings favor a role for white matter network damage in the genesis of cognitive decline in SVD and emphasize the importance of network efficiency as an early marker of SVD progression. Furthermore, this evidence further highlights the potential of this network measures as surrogate marker for SVD in future clinical trials. However, future studies are needed to validate these findings using newer DTI protocols and sophisticated tractography techniques.

## Funding

Dr. AM Tuladhar is a junior staff member of the Dutch Heart Foundation (Grant No 2016T044).

Professor Dr. FE de Leeuw has received the Innovational Research Incentive grant (016-126-351) and the Clinical established investigator Dutch Heart Foundation grant (2014T060).

## CRediT authorship contribution statement

**Esther M. Boot:** Conceptualization, Methodology, Formal analysis, Investigation, Writing - original draft. **Esther MC van Leijsen:** Writing - review & editing. **Mayra I. Bergkamp:** Resources, Writing - review & editing. **Roy P.C. Kessels:** Writing - review & editing. **David G. Norris:** Writing - review & editing. **Frank-Erik de Leeuw:** Project administration, Writing - review & editing. **Anil M. Tuladhar:** Supervision, Writing - review & editing, Funding acquisition.

## Declaration of Competing Interest

The authors declare that they have no known competing financial interests or personal relationships that could have appeared to influence the work reported in this paper.
